# Mapping three decades of air pollution–lung cancer research: trends, hotspots, and networks (1990-2025)

**DOI:** 10.3389/fonc.2025.1698246

**Published:** 2025-12-18

**Authors:** Haixia Fan, Limantian Wang, Lu Zhai, Shudan Deng, Yan Li, Huiyan Niu, Bomeng Zhao, Jie Gao, Xiaoling Gao

**Affiliations:** 1 Department of Sleep Center, First Hospital of Shanxi Medical University, Taiyuan, Shanxi, China; 2 Second College of Clinical Medicine, Shanxi Medical University, Taiyuan, Shanxi, China; 3 Shanxi Medical University, Taiyuan, Shanxi, China; 4 Department of Sleep Center, Second Hospital of Shanxi Medical University, Taiyuan, Shanxi, China

**Keywords:** air pollution, lung cancer, risk factors, PM2.5, bibliometric analysis

## Abstract

**Background:**

The relationship between air pollution and lung cancer has attracted considerable attention from researchers worldwide. To systematically assess the scholarly landscape and pinpoint research fronts, this study employs bibliometric analysis to delineate global trends, collaborative networks, and key publications within this field.

**Methods:**

Publications from 1990 to 2025 were extracted from Web of Science Core Collection and Scopus databases. Bibliometric tools including VOSViewer, Citespace, and Bibliometrix R were used to examine trends, key contributors, research themes, and prominent journals.

**Results:**

Among 4,238 publications, citation rates rose significantly. China produced the most publications, with leading institutions such as Harvard University and the Chinese Academy of Sciences. Key researchers included Lan Q, Rothman N, and Vermeulen R. Major journals were Environmental Health Perspectives and Atmospheric Environment. Frequently used keywords like “Lung Cancer” and “Particulate Matter” indicate core themes, while emerging terms such as “Covid-19” and “Machine Learning” reflect evolving interests.

**Conclusion:**

Fine particulate matter is an established environmental risk factor for lung cancer, and research on polycyclic aromatic hydrocarbons and asbestos remains active. The field has shifted from exposure assessment to mechanistic investigations focusing on oxidative stress, gene expression, and machine learning applications, defining key future research directions.

## 1 Introduction

Lung cancer is one of the most frequently diagnosed malignancies and the leading cause of cancer-related mortality globally, with an estimated 2 million new cases and 1.76 million deaths annually (1, 2). Although tobacco smoking remains the predominant risk factor, the substantial global burden of lung cancer in never-smokers underscores the critical etiological role of alternative factors, particularly ambient air pollution (3). The development of lung cancer in this population likely involves a complex interplay between genetic susceptibility, mediated by germline variants, and environmental exposures, with air pollution being a principal component (4). Ambient air pollution is a complex mixture of particulate matter, gaseous pollutants, and volatile organic compounds emanating from industrial activities, vehicular emissions, energy production, and biomass combustion (5). Among these constituents, fine particulate matter (PM_2.5_) is particularly deleterious due to its capacity to penetrate deep into the respiratory tract and induce pathological processes such as chronic inflammation, oxidative stress, and DNA damage, all of which are key mechanisms in carcinogenesis (6, 7). Similarly, polycyclic aromatic hydrocarbons (PAHs), such as benzopyrene, are established carcinogenic components of air pollution, whose effects are mediated through mechanisms including metabolic activation to DNA-damaging species and the induction of epigenetic alterations (8, 9). As urbanization and industrialization continue to expand worldwide, the burden of lung cancer attributable to air pollution is projected to rise, thereby presenting a pressing public health challenge (10).

Recently, scientific inquiry into the association between air pollution and lung cancer has expanded considerably. This growth has been propelled by advancements in air quality monitoring, the refinement of epidemiological methods, and increasing public awareness of environmental health risks. Epidemiological investigations have employed diverse methodological approaches, which encompass cohort studies, case-control studies, and time-series analyses, to quantify the relationship between long-term air pollutant exposure and lung cancer incidence (11–13). In parallel, molecular studies have elucidated specific genotoxic and epigenetic mechanisms instigated by inhaled pollutants (14). These comprehensive investigations have yielded a substantial body of evidence that spans multiple disciplines, from environmental science and toxicology to oncology, epidemiology, and public health. However, the fragmentation of this knowledge base across disparate scientific fields poses significant challenges for researchers and policymakers who need to synthesize evidence, identify pivotal studies, and monitor emerging trends.

Bibliometric analysis provides a robust quantitative framework for examining the intellectual structure and evolution of scientific fields. It achieves this through the statistical analysis of publications, which yields valuable metrics that cover citations, authorship, keywords, and collaborative networks (15, 16).This methodology enables the identification of research trends, emerging topics, and knowledge gaps within a given domain and has been widely applied across diverse disciplines. Despite the substantial expansion of literature linking air pollution to lung cancer, a comprehensive bibliometric synthesis of this specific field is currently lacking. To address this gap, this study employs bibliometric methods to systematically map the research landscape of air pollution and lung cancer over the past three decades. The study aims to delineate the evolution of key themes, identify influential publications and collaborative networks, and highlight future research directions. Furthermore, the findings are anticipated to inform targeted air quality regulations by identifying the most hazardous pollutants, guide preventive strategies and early detection protocols, and shape public health policies for high-risk populations, thereby contributing to improved lung cancer prevention and control.

## 2 Methods

### 2.1 Eligibility criteria

Eligible studies included those examining the association between air pollution and lung cancer, encompassing human epidemiological research, *in vitro* analyses, and animal models. Specifically, for the bibliometric analysis, only primary research and review articles were included. The exclusion criteria comprised non-English publications, conference abstracts, editorials, letters, books, book chapters, as well as studies where the air pollution-lung cancer relationship was not a central focus. To minimize selection bias, two reviewers independently screened studies and applied the eligibility criteria. Subsequently, any disagreements were resolved through consensus or, if necessary, by consulting a third reviewer.

### 2.2 Search strategy

This study conducted an integrated search across both the Web of Science Core Collection (WoSCC) and Scopus databases to ensure a comprehensive bibliometric analysis. This methodology aims to elucidate evolving research trends and intellectual landscapes by leveraging the complementary nature of these databases. Specifically, WoSCC provides rigorous journal selection and reliable citation tracking, which facilitates the assessment of scholarly impact. In contrast, Scopus offers extensive interdisciplinary coverage and advanced analytical tools, enabling a holistic overview of the research field (17, 18).

**Figure 1:**
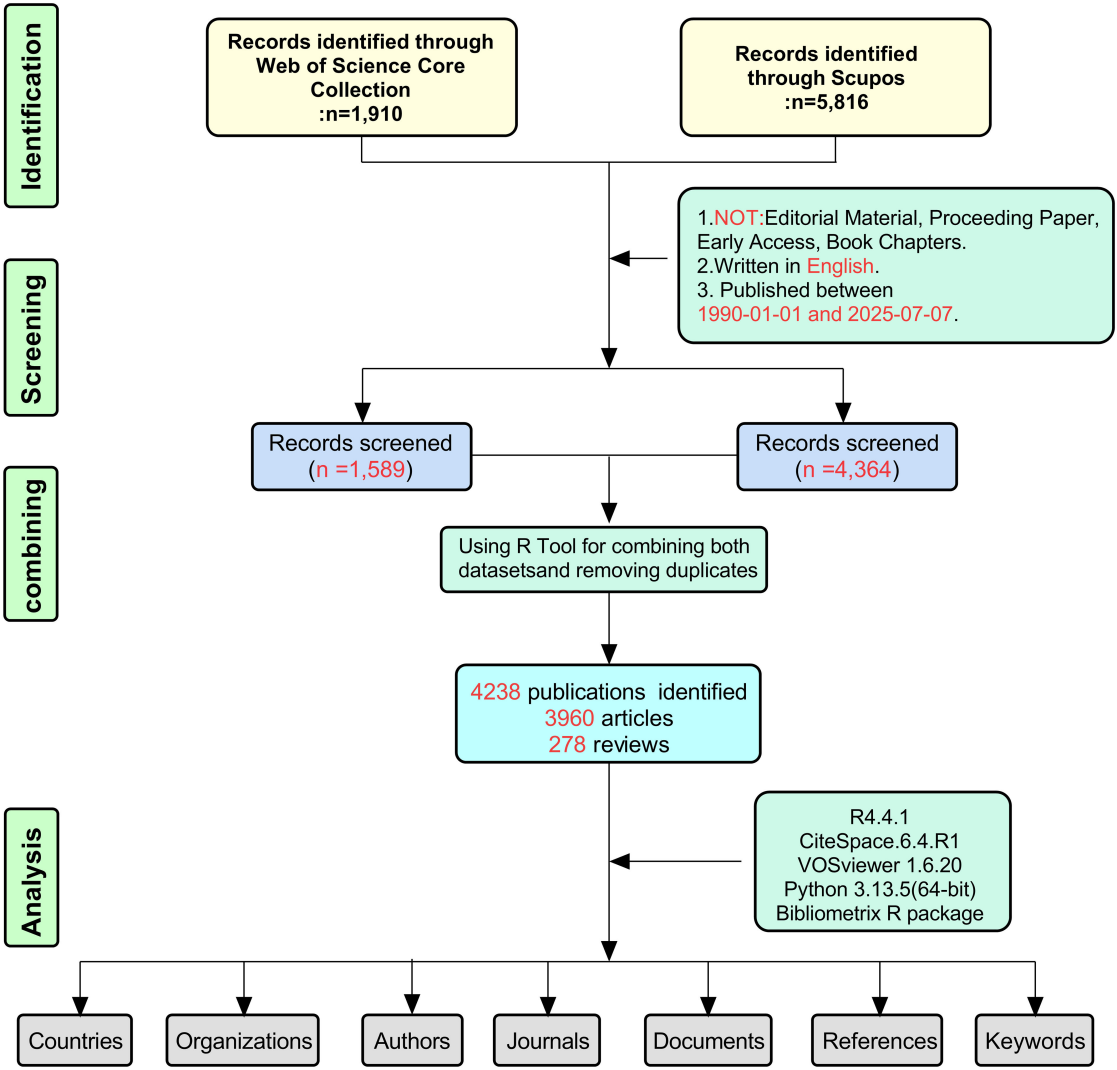
The flowchart for literature search, selection and analysis.

The data retrieval followed a systematic protocol, as detailed in **Figure 1**. A comprehensive search of the WoSCC and Scopus databases was conducted on July 7, 2025. This search employed predefined keywords to query publication titles, abstracts, and author keywords. For the selection of relevant terms, we reviewed several previous publications in the literature (19, 20). Search terms for air pollution: (“Air Pollution*” OR “Pollution, Air” OR “Air Quality” OR “Air Pollutant*” OR “Pollutants, Air” OR “Pollutant, Air” OR “Environmental Pollution” OR “Pollution, Environmental” OR “Air Pollutants, Environmental” OR “Environmental Pollutants, Air” OR “Air Environmental Pollutants” OR “Pollutants, Air Environmental” OR “Environmental Air Pollutants”). Search terms for lung cancer: (“Neoplasm, Lung” OR “Neoplasms, Pulmonary” OR “Neoplasm, Pulmonary” OR “Lung Neoplasm” OR “Lung Neoplasms” OR “Neoplasm, Lung” OR “Lung Cancer” OR “Cancer, Lung” OR “Cancers, Lung” OR “Lung Cancers” OR “Cancer of Lung” OR “Pulmonary Cancer” OR “Cancer, Pulmonary” OR “Cancers, Pulmonary” OR “Pulmonary Cancers” OR “Cancer of the Lung”). The search strategy combined the two sets of terms using the Boolean operator “AND” to ensure that retrieved publications addressed both air pollution and lung cancer (**Supplementary Document**).

This initial search yielded 1,910 records from WoSCC and 5,816 from Scopus. The dataset was then refined by excluding non-primary and non-English literature, including document types such as proceeding papers, corrections, news items, book chapters, and letters. Only English-language articles and reviews published between 1990 and 2025 were retained for subsequent analysis (21, 22). Following these exclusion steps, 1,589 documents from WoSCC and 4,364 from Scopus remained eligible (**Figure 1**).

The datasets obtained from the WoSCC and Scopus were merged using the bibliometrix R package. A multi-step matching process was implemented to identify duplicate records, primarily relying on titles, authors, publication years, and digital object identifiers (DOIs). For records lacking a DOI, title and first author name served as secondary matching criteria. All potential duplicates identified through this automated screening underwent manual verification to eliminate double-counting (23). This deduplication process resulted in a final corpus of 4,238 unique publications for analysis, comprising 3,960 articles and 278 reviews. The combined dataset incorporated all 1,589 records from WoSCC and an additional 2,649 unique records from Scopus (**Figure 1**). Collectively, these steps effectively mitigated duplication bias and ensured data integrity.

### 2.3 Study selection

Two reviewers independently screened titles and abstracts, followed by full-text evaluation against eligibility criteria. Discrepancies were resolved by discussion. After duplicate removal, 4,238 unique studies were included for further analysis.

### 2.4 Data extraction

For each eligible study, data were extracted on publication year, study type, population/sample, specific air pollutants investigated, exposure assessment methods, and key findings regarding lung cancer risk or mechanisms. For the bibliometric analysis, standard metadata, including authors, institutions, journals, citations, and keywords, were collected. To ensure the accuracy and consistency of data extraction, the process was conducted independently by two reviewers, with subsequent cross-checking to resolve any discrepancies.

### 2.5 Bibliometric analysis

Bibliometric and analyses were conducted using multiple complementary tools to ensure robustness. This methodology was implemented as described in the relevant literature (24). CiteSpace (6.4.R1, 64-bit Advanced Edition) was employed to generate knowledge maps of authors, institutions, and keywords, with a one-year time slice spanning 1990–2025 (25). Author and institution nodes were limited to the top 25 per slice, while keyword networks were pruned with pathfinder and merged techniques to highlight major thematic clusters. VOSviewer (1.6.20) was applied with full counting to construct visual representations of collaborative networks, capturing co-authorship and institutional partnerships (26). The R package bibliometrix (https://www.bibliometrix.org), was used for historiographic mapping and calculation of bibliometric indicators, including g-index, h-index, number of citations (NC), and number of publications (NP), thereby quantifying research productivity and impact. To support data management, Microsoft Excel 2021(Version 16. 48) was used to organize and clean initial datasets, while the Online Analysis Platform of Literature Metrology (https://bibliometric.com/) provided intuitive visualization of citation dynamics (27, 28). Together, these tools enabled a comprehensive evaluation of publication trends, influential contributors, collaborative structures, and emerging hotspots in air pollution and lung cancer research.

## 3 Results

### 3.1 Annual publications trends

**Figure 2:**
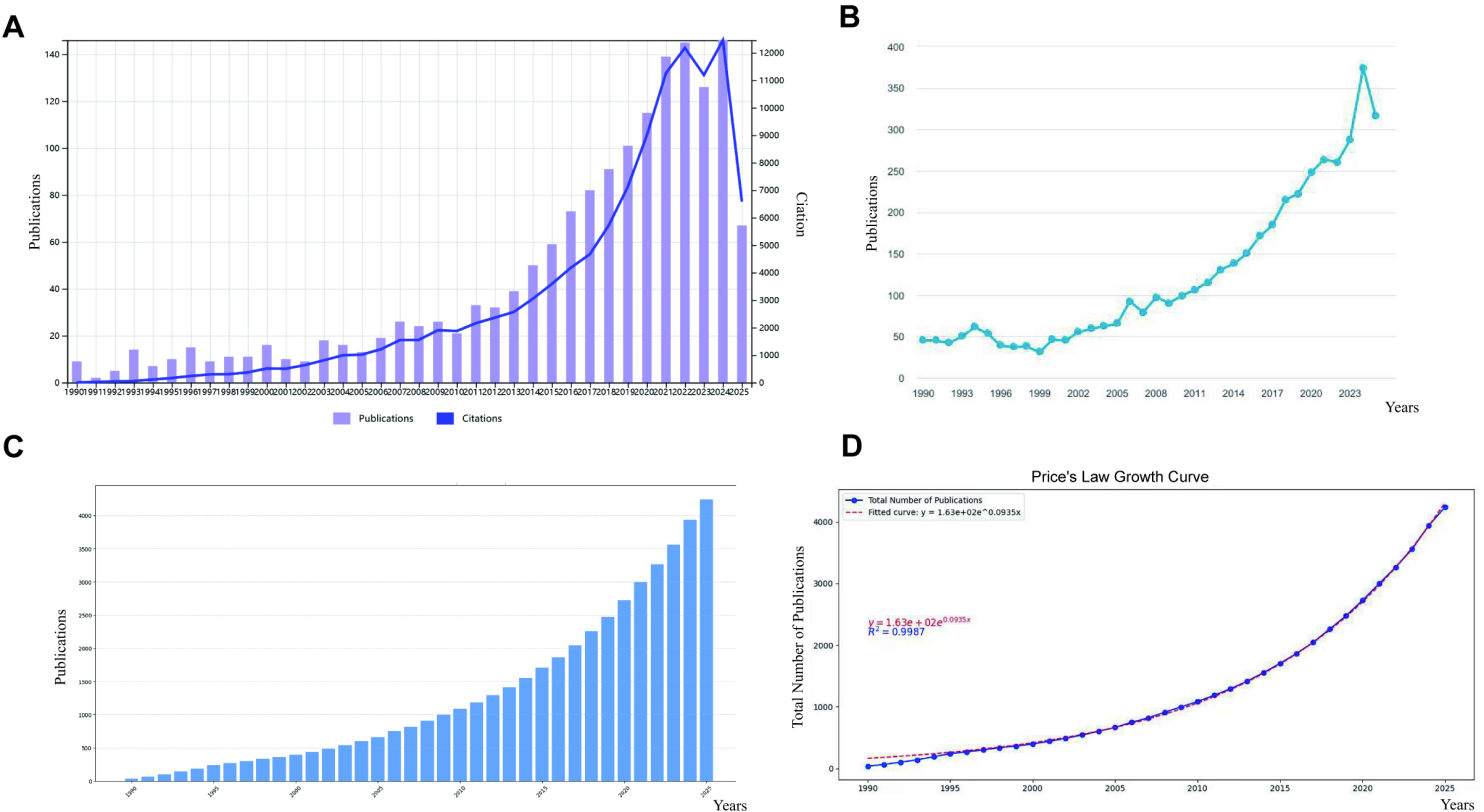
Overview of publications and citations on air pollution and lung cancer (1990–2025). **(A)** Trends in annual scientific output in WoSCC with respect to Publications and Citations. **(B)** Trends in annual scientific output in Scopus with respect to publications and citations. **(C)** Cumulative number of publications. **(D)** Price’s Law of cumulative growth curve fitting analysis.

Our analysis encompassed the period from 1990 to 2025, examining a corpus of 4,238 documents that provided a comprehensive overview of nearly three decades of scholarly activity. As illustrated in **Figure 2A**, the WoSCC database revealed parallel trajectories for both publications and citations, with both metrics experiencing significant growth over time, reaching their maximum around 2023, and subsequently decreasing. Similarly, Scopus data (**Figure 2B**) showed a steady increase in annual publications, with a notable acceleration after 2015. This upward trend culminated in a peak in 2023, followed by a subsequent decline. The analysis of publication trends revealed a steady increase from 35 publications in 1990 to 302 in 2025. Notably, the most rapid expansion occurred in recent years, with a peak of 380 articles recorded in 2024, reflecting a robust expansion in research activity (**Figure 2C**). It is important to note that the apparent declines observed after 2023 in **Figures 2A, B**, and the value for 2025 in **Figure 2C** are likely artifacts of the “indexing delay” inherent in bibliographic databases, where recent publications are not yet fully captured, rather than representing an actual decrease in scholarly output. To evaluate the projected versus actual publication trends, we applied Price’s Law growth curve (**Figure 2D**), which demonstrated a close alignment with an exponential growth pattern. A logistic regression model y = 1.63e+02e^ (0.0935x) was employed to further analyze the growth trajectory, indicating a moderate fit.

### 3.2 Distributions of countries/regions

**Figure 3:**
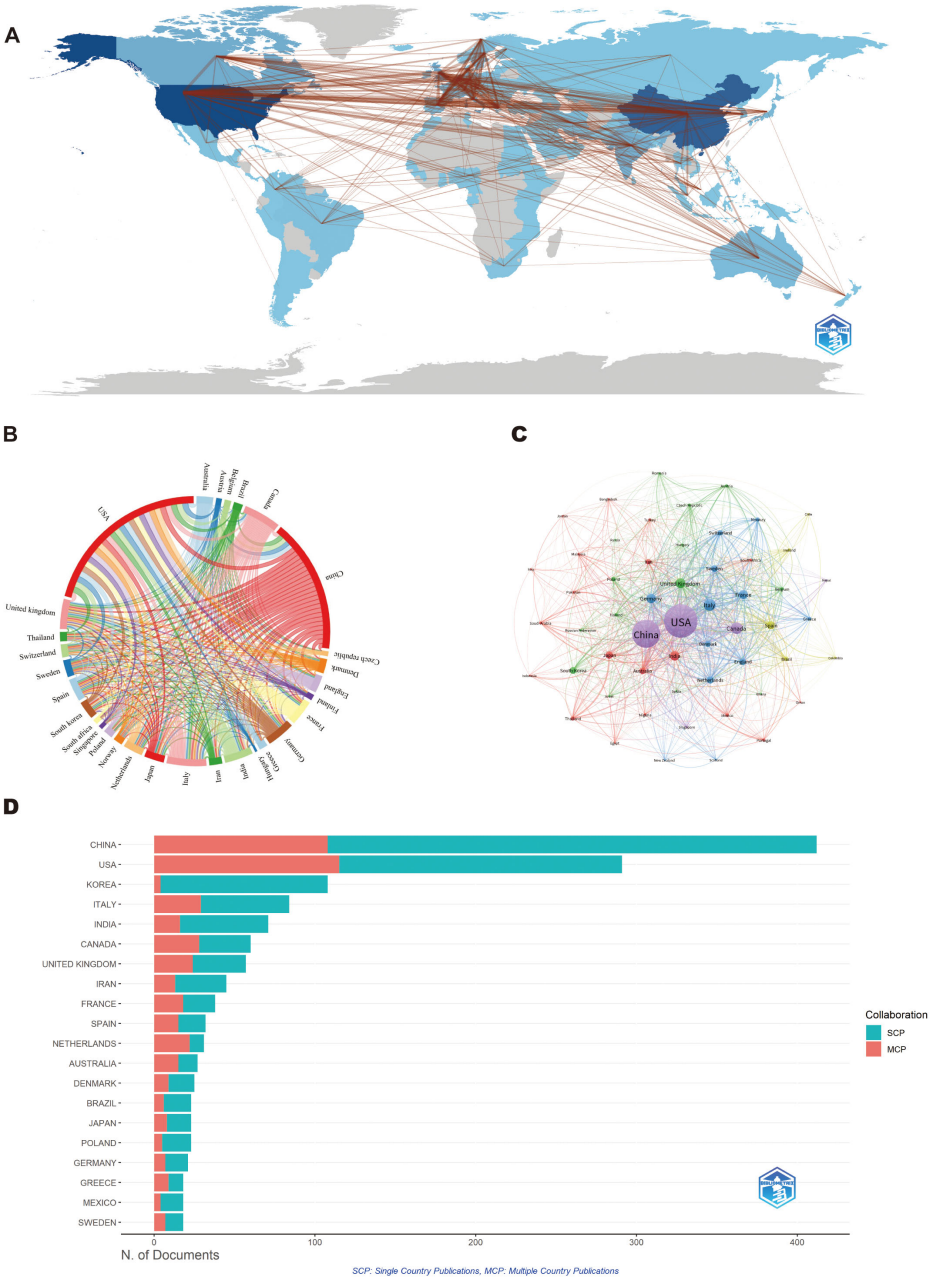
Global trends and collaboration networks in scientific publications on air pollution and lung cancer. **(A)** Country/Region Collaboration Map. Darker shades of blue indicate higher collaboration rates, and the width of the link lines represents the strength of collaboration between two countries. **(B)** Network Map of National Research Output and Cooperative Relations. **(C)** Co-Country Network Analysis The network visualization displays the co-citation relationships between different countries in Air Pollution and lung cancer research, where nodes represent individual countries and edges indicate co-citation patterns between publications from these nations. **(D)** The top 10 countries responsible for the number of studies (Bibliometrix). MCP: Multiple-Country Publications; SCP: Single-Country Publications; OALM: Online Analysis Platform of Literature Metrology.

**Figure 3A** illustrated the geographical distribution of research collaborations, identifying the United States, Europe, and key Asian regions as primary hubs, with the United States serving as the central node. The network exhibited dense cross-continental linkages, reflecting robust multinational cooperation, particularly among North America, Europe, and East Asia. **Figure 3B** confirmed that. As shown in **Figure 3C**, co-authorship networks reveal a highly interconnected global research community. The United States and China emerged as the most prominent hubs, characterized by extensive international linkages. Europe forms a densely integrated cluster, led by the United Kingdom, Germany, France, and Italy, which effectively bridges North American and Asian research activities. Other important connectors include South Korea, Japan, India, and Australia, while smaller nations including Switzerland, Spain, and the Netherlands maintain strong connectivity, enhancing the overall robustness of the network. **Figure 3D** provided a comparative analysis of national publication output, distinguishing between single-country publications (SCP, blue) and multi-country publications (MCP, red). China led in total output with 412 articles (9.7%), followed by the United States with 291 publications (6.9%). The United States demonstrated stronger international engagement (MCP 39.5%), whereas China’s publications were more domestically oriented (MCP 26.2%). Beyond these two leading nations, significant contributions originate from other Asian countries such as South Korea and India, alongside several European nations including Italy, the United Kingdom, and Germany. The Netherlands stands out for its exceptionally high degree of international collaboration. Additional meaningful contributions come from countries across Oceania, South America, and other global regions (**Supplementary Table 1**).

### 3.3 Distribution by institutions

**Figure 4:**
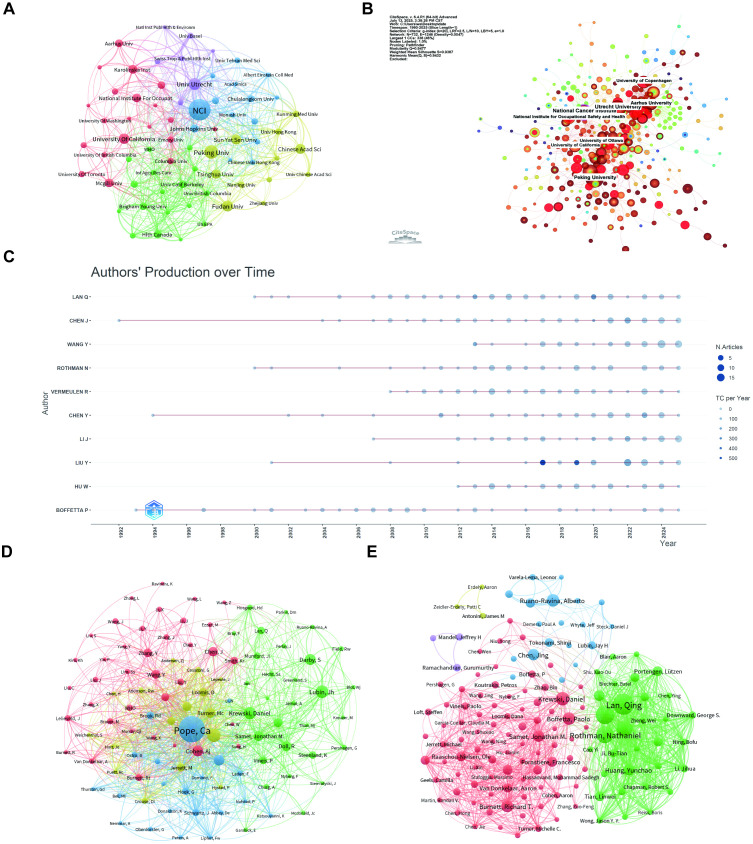
Institutional contributions and collaboration networks and co-authorship and co-cited authorship networks on air pollution and lung cancer. **(A)** Institutional Ccollaboration network (VOSviewer). Node size corresponds to collaboration extent, and colors denote distinct regional or institutional clusters. **(B)** Research institution network (CiteSpace). Nodes represent institutions, with size reflecting publication volume and line thickness indicating collaboration strength. **(C)** Annual Publication Trends of Top 10 Authors. **(D)**Co-cited authorship network (VOSviewer): Nodes, connected by collaborative publications, have edge thickness proportional to joint works. **(E)** Clusters, marked by colors, reveal research groups. Co-authorship network (VOSviewer): Nodes represent authors, sized by publication count. Colors indicate collaboration clusters.

As demonstrated in **Supplementary Table 2**, Harvard University, Utrecht University, and the Chinese Academy of Sciences accounted for the highest number of publications, with 146, 115, and 106 articles, respectively. Furthermore, **Figure 4A**, generated by VOSviewer, highlighted the National Cancer Institute (NCI) as the most prominent contributor in terms of research relevance, followed closely by Peking University. Notably, NCI, Peking University, and the University of California system emerge as pivotal players in this network, fostering robust research partnerships. This was evidenced by the dense clusters of red and green nodes, which reflect strong collaborative ties. The collaboration network extends globally, with significant contributions from other institutions such as Fudan University and the Karolinska Institute. The CiteSpace map further highlighted three distinct regional collaboration hubs. A dense brown-red cluster centered around Peking University underscores China’s high research output and strong domestic linkages. In the upper right quadrant, a cohesive European network was formed by red-orange nodes surrounding Utrecht University, Aarhus University, and Copenhagen. Meanwhile, the upper left quadrant revealed tightly grouped North American centers, including NCI, the University of California system, Ottawa, and NIOSH, which are strongly connected to both European and Chinese hubs. Node size reflects publication volume, while the thickness of intercontinental edges emphasizes the truly global nature of these partnerships (**Figure 4B**). This multi-scale network not only highlights the foundational role of core institutions but also illustrates the intricate interconnections that define the global research landscape.

### 3.4 Distributions of authors and co-cited authors

A total of 16,823 authors contributed to publications in this field. Among them, only 272 authored single-authored works, underscoring the highly collaborative nature of research in this domain. Furthermore, the average number of authors per publication was six, and 13.1% of publications involved international co-authorship (**Supplementary Table 3**). **Figure 4C** presented the temporal publication profiles of the ten most prolific authors, showcasing their multi-decadal engagement and evolving research impact. Notably, authors such as Boffetta P and Chen J first emerged in the 1990s and maintained a consistent publication trajectory up to 2025. In contrast, Lan Q and Rothman N began their publishing careers around 2000, demonstrating increasing productivity and citation peaks during the mid-2010s. More recent entrants, including Li J and Vermeulen R, launched their contributions in the 2010s and rapidly achieved high productivity and citation rates by 2020, which underscored their growing influence within the field.

Complementing these temporal analyses, **Figures 4D, E** offered insights into the collaborative and intellectual structures of the field through co-authorship and co-citation network analyses. **Figure 4D** showed a large, densely connected co-authorship network in which Pope C.A. occupied the central and most influential position, collaborating extensively with authors such as Krewski D., Burnett R.T., Jerrett M., Thurston G.D., Dockery D.W., Samet J.M., and Turner M.C.; the network is divided into color-coded clusters representing collaboration communities, with Pope C.A.’s group forming the largest and most interconnected cluster, reflecting strong interdisciplinary partnerships and high citation impact across environmental epidemiology and air pollution research. **Figure 4E** illustrated more clearly defined author clusters, with one major community led by Lan Qings, strongly connected to collaborators such as Rothman N., Boffetta P., Vermeulen R., Liu Y., Hu W., Chen J., and Wang Y., while another major cluster centered around Boffetta P., linking densely with authors including Peters A., Braun S., Ramanzini J., and Zhang Y.; this structure highlights the existence of distinct, specialized research groups in molecular epidemiology, occupational exposure, and air pollution-related carcinogenesis with intensive internal collaboration. The networks revealed that a small number of influential clusters—centered around Pope C.A. and Lan Qings—dominate research on lung cancer and air pollution through dense, long-term co-authorship networks.

### 3.5 Journals and co-journals

**Figure 5:**
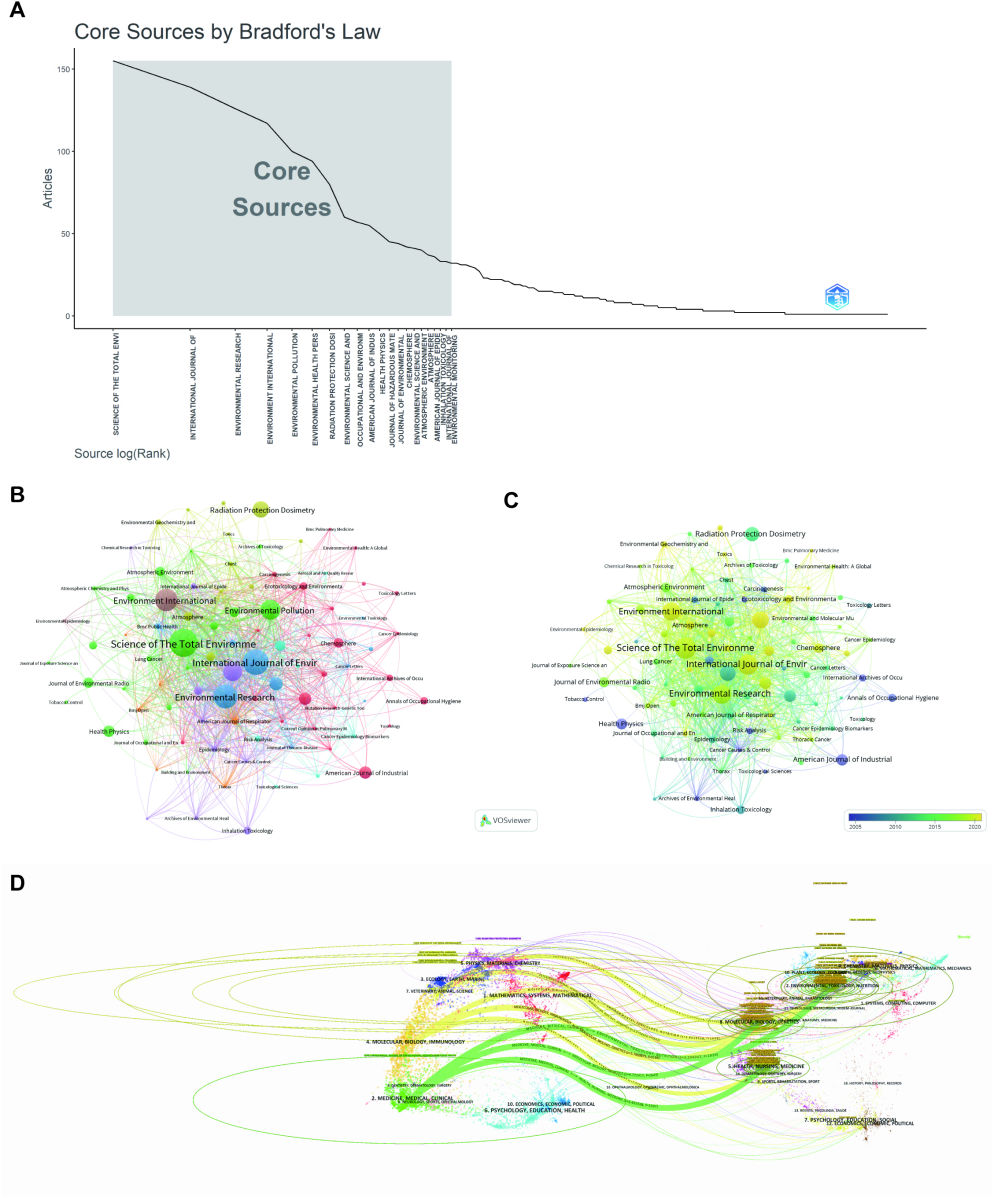
Comprehensive analysis of journals and citation networks related to air pollution and lung cancer. **(A)** Bradford’s Law analysis (Bibliometrix): Identifies core journals based on publication distribution. **(B)** Journal Clustering Analysis and **(C)** Co-Journal Clustering Analysis - Each circle represents a journal. Circle size corresponds to the strength of connections, citation counts, and other metrics. Circle colors indicate the cluster affiliation. **(D)** Journal Dual-Map Overlay Analysis: Dual-map visualization showing citing and cited journal relationships, with lines indicating citation flows and knowledge transfer.

The selection of the top ten most influential journals was based on multiple criteria, including publication volume, citation impact, and journal impact factors. A small number of multidisciplinary journals dominate the field’s output, with *Science of the Total Environment* leading with over 150 publications, followed by *International Journal of Environmental Research* and *Public Health and Environmental Research*, each exceeding 120 articles. *Environmental International* and *Environmental Pollution* complete this central group, collectively accounting for approximately half of all papers (**Table 1**). A secondary tier, including *Environmental Health Perspectives*, Radiation Protection Dosimetry, and *Environmental Science and Pollution Research*, contributes a steady stream of studies, while a long tail of specialized outlets publishes progressively fewer articles (**Figure 5A**).

**Table 1:** Top 10 journals by influence rank according to bibliometrix analysis (1990-2025).

Rank	Source	h_index	g_index	m_index	TC	NP	PY_start	IF	JCR
1	ENVIRONMENTAL HEALTH PERSPECTIVES	54	94	1.688	13765	94	1994	9.8	Q1
2	ENVIRONMENTAL RESEARCH	45	77	1.286	6184	126	1991	7.7	Q2
3	SCIENCE OF THE TOTAL ENVIRONMENT	44	71	1.333	5804	155	1993	8	Q2
4	ENVIRONMENT INTERNATIONAL	40	77	1.111	6171	117	1990	9.1	Q1
5	INTERNATIONAL JOURNAL OF ENVIRONMENTAL RESEARCH AND PUBLIC HEALTH	32	60	1.778	4267	139	2008	3.5	Q4
6	ENVIRONMENTAL POLLUTION	31	83	1	6927	100	1995	7.3	Q2
7	OCCUPATIONAL AND ENVIRONMENTAL MEDICINE	29	45	0.935	2214	57	1995	3.1	Q2
8	AMERICAN JOURNAL OF EPIDEMIOLOGY	26	36	0.722	2772	36	1990	4.8	Q2
9	AMERICAN JOURNAL OF INDUSTRIAL MEDICINE	26	47	0.743	2315	55	1991	3.1	Q3
10	ATMOSPHERIC ENVIRONMENT	24	40	1.412	1776	40	2009	3.7	Q3

IF, Impact Factor; JCR, Journal Citation Reports.

**Figures 5B** and 5C presented the co-citation networks of these journals, offering insights into inter-journal relationships. The network visualization (**Figure 5B**) illustrated that *Science of the Total Environment* and *Environmental Research* serve as pivotal nodes, heavily interconnected with other leading such as International Journal of *Environmental Research and Public Health*, *Environmental International*, and *Environmental Pollution*. Clusters of distinct colors—green for atmospheric and air-quality research, red for occupational and industrial health, blue/purple for inhalation and toxicological sciences, and yellow for molecular toxicology—indicate thematic groupings, suggesting that journals within each cluster frequently co-publish related topics and thereby form discrete research subfields. Furthermore, **Figure 5C** provided a temporal overlay of these co-citation relationships, showing the evolution of the network from 2005 through 2020. The visualization revealed a notable expansion of interconnectivity beginning around 2006, signaling growing interdisciplinary collaboration. The color gradient, shifting from dark blue (circa 2005) through green (circa 2015) to bright yellow (circa 2020), highlighted a recent concentration of co-citations in journals like *Environment International* and *Environmental and Molecular Mutagenesis*, reinforcing the trend toward intensified collaboration in recent years. This dynamic network structure underscored the emergence of increasingly integrated research themes, pointing to an evolving and more interconnected landscape in environmental and toxicological sciences.

Additionally, the dual-map overlay generated by CiteSpace (**Figure 5D**) delineated interdisciplinary citation patterns and knowledge flows within air pollution and lung cancer research. Analysis of the overlay revealed that citing journals were predominantly clustered within medicine, molecular biology, environmental science, and public health. This distribution reflects the field’s multifactorial approach to connecting pollutant exposure with oncogenic outcomes. Furthermore, citation trajectories converged predominantly in journals focused on molecular biology and genetics, environmental toxicology, and clinical medicine, indicating a foundational reliance on mechanistic, toxicological, and epidemiological evidence.

### 3.6 References and articles

The top 25 most cited documents are presented in **Table 2**. Analysis of these seminal works revealed the evolution of research hotspots in the air pollution and lung cancer field, beginning with foundational cohort studies that established mortality links (Dockery et al., 1993; Pope et al., 2002) (29, 30) and progressing toward more nuanced investigations. Current research efforts focus on three main areas: quantifying the carcinogenic effects of specific pollutants including PM_2.5_ across global populations (Burnett et al., 2014) (31), elucidating biological mechanisms such as oxidative stress (Lepuele et al., 2012) (32), and refining exposure assessment through advanced modeling techniques. Recent studies also emphasize gene-environment interactions (Lan et al., 2002) (33) and the contribution of emerging pollution sources to lung adenocarcinoma. Collectively, these studies highlight a paradigm shift from establishing general associations to developing mechanistic understanding and advancing personalized risk assessment.

**Table 2:** Top 25 most cited publications based on bibliometrix analysis (1990-2025).

Rank	Document	Year	DOI	Local Citations	Global citations	LC/GC ratio (%)	Normalized local citations	Normalized global citations
1	POPE CA, 2002, JAMA-J AM MED ASSOC	2002	10.1001/jama.287.9.1132	278	6127	4.54	31.55	30.48
2	RAASCHOU-NIELSEN O, 2013, LANCET ONCOL	2013	10.1016/S1470-2045(13)70279-1	174	1153	15.09	32.02	10.76
3	DOCKERY DW, 1993, NEW ENGL J MED	1993	10.1056/NEJM199312093292401	139	5411	2.57	31.77	31.99
4	BURNETT RT, 2014, ENVIRON HEALTH PERSP	2014	10.1289/ehp.1307049	110	1435	7.67	33.17	18.08
5	TURNER MC, 2011, AM J RESP CRIT CARE	2011	10.1164/rccm.201106-1011OC	80	420	19.05	17.58	4.38
6	LAN Q, 2002, J NATL CANCER I	2002	10.1093/jnci/94.11.826	74	212	34.91	8.40	1.05
7	LADEN F, 2006, AM J RESP CRIT CARE	2006	10.1164/rccm.200503-443OC	73	1074	6.80	30.14	7.32
8	LEPEULE J, 2012, ENVIRON HEALTH PERSP	2012	10.1289/ehp.1104660	70	747	9.37	26.85	13.49
9	CESARONI G, 2013, ENVIRON HEALTH PERSP	2013	10.1289/ehp.1205862	68	419	16.23	12.51	3.91
10	ABBEY DE, 1999, AM J RESP CRIT CARE	1999	10.1164/ajrccm.159.2.9806020	61	430	14.19	16.18	6.28
11	NYBERG F, 2000, EPIDEMIOLOGY	2000	10.1097/00001648-200009000-00002	59	314	18.79	12.67	3.03
12	CAO J, 2011, J HAZARD MATER	2011	10.1016/j.jhazmat.2010.12.036	58	310	18.71	12.75	3.23
13	BEESON WL, 1998, ENVIRON HEALTH PERSP	1998	10.2307/3434125	57	182	31.32	20.94	3.94
14	POPE CA, 2011, ENVIRON HEALTH PERSP	2011	10.1289/ehp.1103639	55	557	9.87	12.09	5.80
15	HYSTAD P, 2013, EPIDEMIOLOGY	2013	10.1097/EDE.0b013e3182949ae7	54	145	37.24	9.94	1.35
16	HOEK G, 2002, LANCET	2002	10.1016/S0140-6736(02)11280-3	53	1135	4.67	6.01	5.65
17	KATANODA K, 2011, J EPIDEMIOL	2011	10.2188/jea.JE20100098	53	201	26.37	11.65	2.09
18	PUETT RC, 2014, ENVIRON HEALTH PERSP	2014	10.1289/ehp.1307490	52	129	40.31	15.68	1.63
19	BEELEN R, 2008, EPIDEMIOLOGY	2008	10.1097/EDE.0b013e318181b3ca	50	169	29.59	14.65	1.30
20	CHEN J, 2020, ENVIRON INT	2020	10.1016/j.envint.2020.105974	48	636	7.55	25.73	9.40
21	NAFSTAD P, 2003, THORAX	2003	10.1136/thorax.58.12.1071	47	166	28.31	25.13	1.08
22	BEELEN R, 2008, ENVIRON HEALTH PERSP	2008	10.1289/ehp.10767	47	473	9.94	13.77	3.63
23	TURNER MC, 2020, CA-CANCER J CLIN	2020	10.3322/caac.21632	47	522	9.00	25.19	7.72
24	SUN S, 2007, NAT REV CANCER	2007	10.1038/nrc2190	46	1327	3.47	15.85	13.23
25	HAMRA GB, 2015, ENVIRON HEALTH PERSP	2015	10.1289/ehp.1408882	46	289	15.92	19.46	4.56

DOI, Digital Object Identifier.

**Figure 6:**
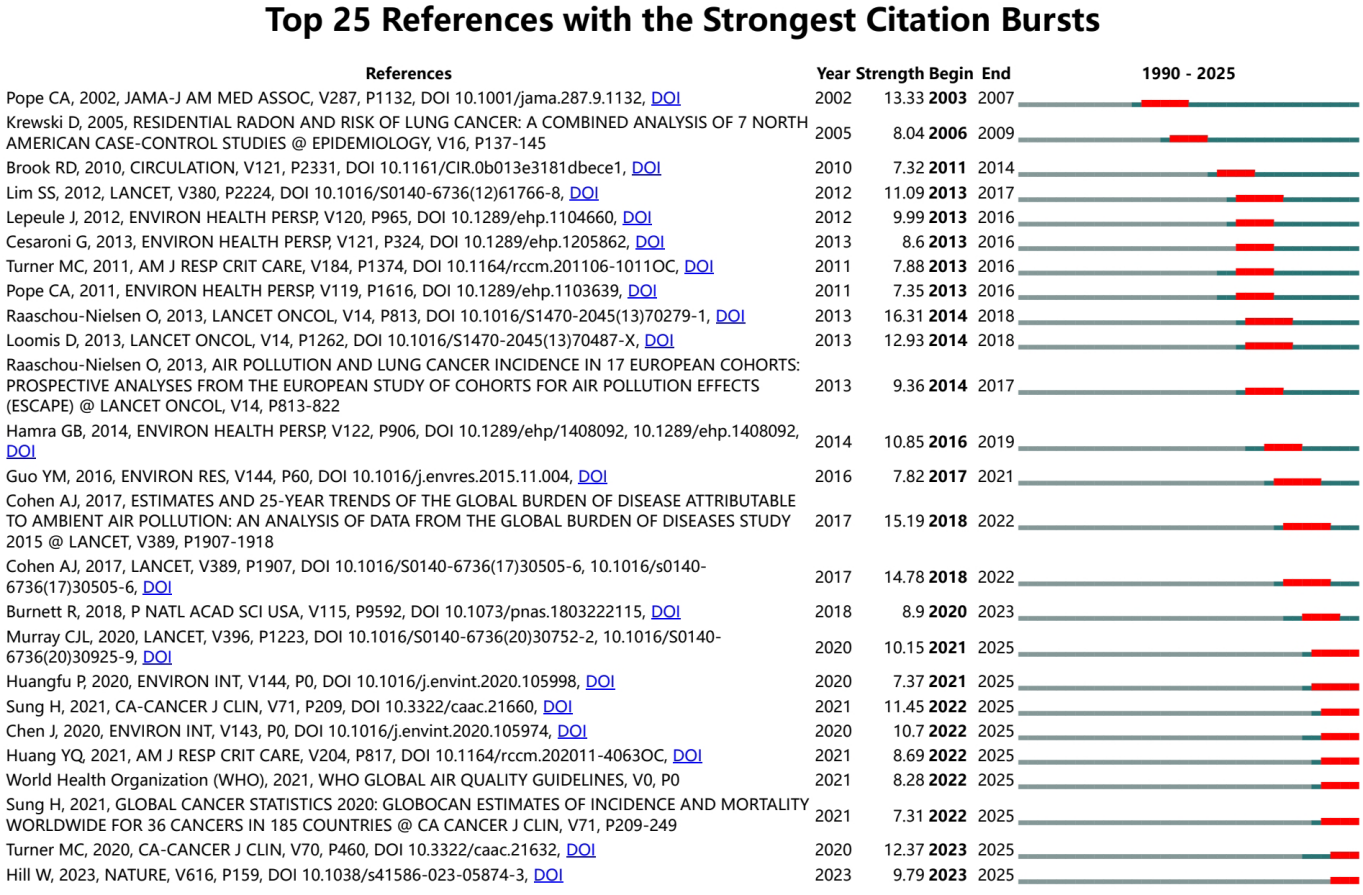
Top 25 references with strongest citation bursts in air pollution and lung cancer. The burst timeline (CiteSpace) shows the periods during which these bursts occurred, with red segments indicating the time intervals when each reference received heightened attention.

**Figure 6** depicted the top 25 references with the strongest citation bursts, arranged chronologically to illustrate evolving research emphases. Notably, Raaschou-Nielsen’s 2013 Lancet Oncology paper (34) exhibited the highest burst strength (16. 31) from 2014 to 2018, underscoring its transformative impact on linking ambient air pollution to lung cancer incidence. Earlier, Pope et al.’s 2002 JAMA study (30) showed a robust burst (13. 33) from 2003 to 2007, reflecting the rapid uptake of evidence on particulate-related mortality. Subsequently, bursts such as Lim SS 2012 (35)(Lancet; strength 11. 09, burst 2013–2017) and Lepeule J 2012 (32) (EHP; strength 9. 99, burst 2013–2016) highlight periods of heightened focus on global burden assessments and long-term exposure effects. More recent surges such as Murray CJL 2020 (36) (Lancet; strength 10. 15, 2021–2025), indicate growing attention to integrated disease burden quantification and policy translation. This temporal pattern of citation bursts reveals shifting research frontiers, from establishing causality to refining risk estimates and informing international air quality guidelines.

### 3.7 Keyword analysis

**Figure 7:**
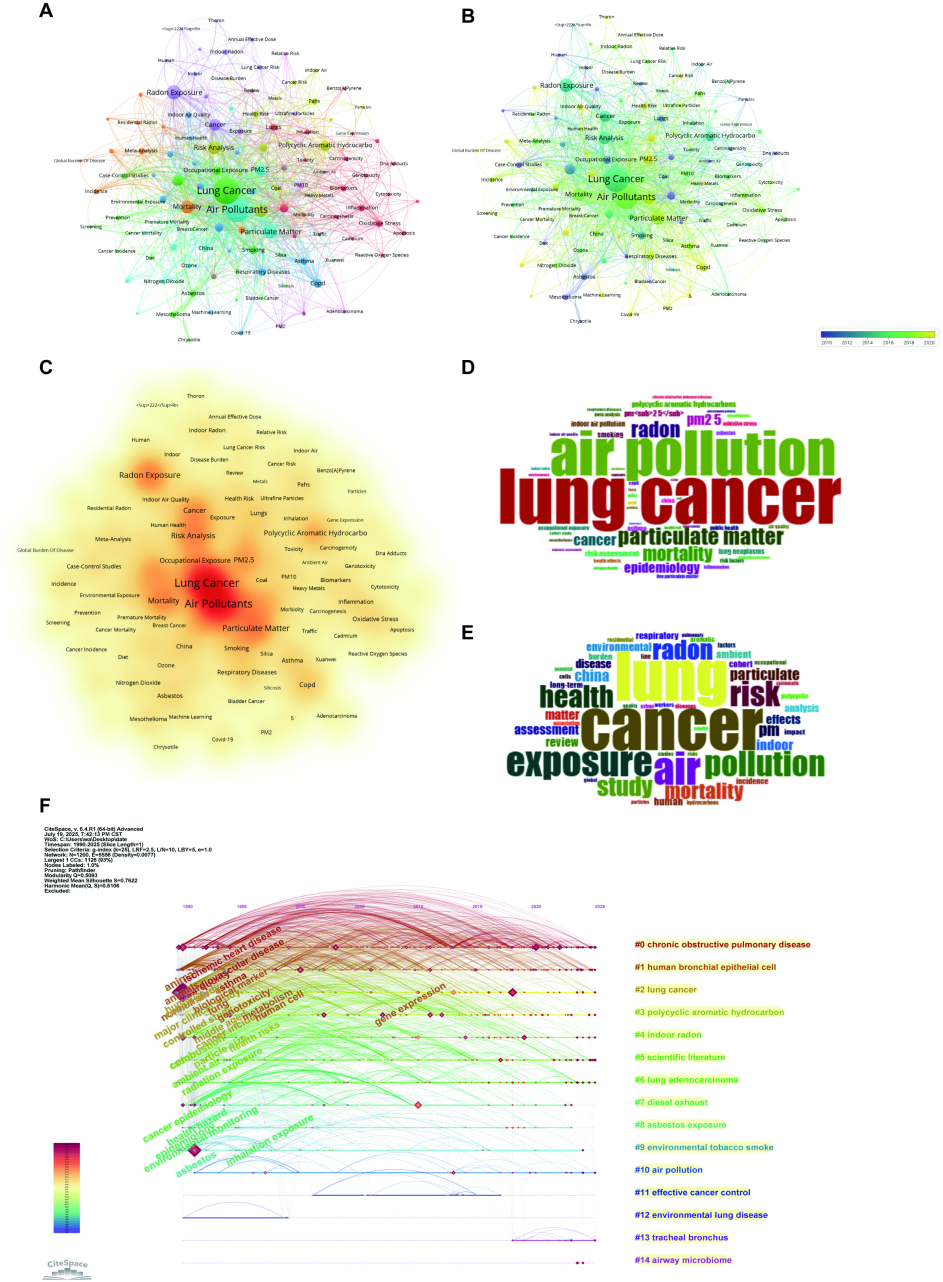
Keywords mapping of air pollution and lung cancer. **(A)** Keywords co-occurrence network (VOSviewer): Nodes represent keywords, sized by occurrence frequency (larger = more frequent), with edges indicating co-occurrence and colors denoting thematic clusters. **(B)** Keywords timeline (VOSviewer): Nodes, sized by frequency and colored by cluster, illustrate the temporal evolution of research topics. **(C)** Density Visualization of Keyword Co-occurrence. Red areas represent regions of high keyword density, suggesting the core topics where research efforts are most concentrated. **(D)** Author keywords word cloud (Bibliometrix): Word size reflects frequency, highlighting prevalent author-assigned terms. **(E)** Title keywords word cloud (Bibliometrix): Word size indicates frequency, emphasizing key title-derived themes. **(F)** Timeline of Keyword Evolution of Air Pollution and Lung Cancer.

**Figure 7A** depicted the complex interconnections among research themes in the field of environmental exposure and lung cancer, as visualized through a keyword co-occurrence network. Notably, central nodes such as “Lung Cancer,” “Air Pollutants,” “Particulate Matter,” and “PM2. 5” dominate the network, underscoring their critical roles in this research area. Furthermore, other prominent clusters include themes related to “Radon Exposure,” “Risk Analysis,” “PAHs,” and “Oxidative Stress,” which highlight parallel investigations into carcinogenic mechanisms, pollutant types, and risk assessment methodologies. The word cloud analysis (**Figures 7D, E**) confirmed that.

**Figure 7B** showed the temporal progression of keyword evolution, revealing distinct research trends over the decade. Earlier studies, represented in blue-purple hues, primarily focused on foundational environmental health topics such as “Radon Exposure,” “Asbestos,” “Nitrogen Dioxide,” and “Cancer Incidence.” In contrast, recent research trends, marked in yellow, have shifted toward emerging keywords like “Covid-19,” “Machine Learning,” “Oxidative Stress,” and “Gene Expression.” **Figure 7C** presented a density visualization that emphasizes the most intensively studied terms. The warmest color gradients (red and orange) cluster around core concepts as “Lung Cancer,” “Air Pollutants,” and “Particulate Matter,” underscoring their prominence in the literature. Surrounding these central themes are moderately dense regions representing topics like “Risk Analysis,” “Radon Exposure,” and “PAHs,” indicating their significant but secondary relevance. In contrast, peripheral terms such as “Covid-19,” “Machine Learning,” and “Adenocarcinoma” appear in lower-density areas, suggesting their status as emerging or niche research directions.

**Figure 7F**, generated using CiteSpace, delineated a coherent intellectual structure comprising 15 thematic clusters. Cluster #0 (chronic obstructive pulmonary disease) constituted a major and persistent research domain, directly linking respiratory disease progression to pollutant exposure. The mechanistic core was anchored by Cluster #1 (human bronchial epithelial cell), which connected laboratory models to specific toxicants, including PAHs in Cluster #3 (polycyclic aromatic hydrocarbon) and diesel exhaust in Cluster #7 (diesel exhaust). Cluster #2 (lung cancer) functioned as the central hub, integrating several exposure-focused clusters, notably Cluster #6 (lung adenocarcinoma), Cluster #4 (indoor radon), and Cluster #8 (asbestos exposure). Concurrently, Cluster #10 (air pollution) and Cluster #9 (environmental tobacco smoke) captured major environmental exposure themes and formed a combustion-related network with Cluster #3 (polycyclic aromatic hydrocarbon) and Cluster #7 (diesel exhaust). Cluster #5 (scientific literature) encompassed methodological studies and connected peripherally to most domains. In contrast, Cluster #11 (effective cancer control) translated research findings into public health applications. Cluster #12 (environmental lung disease) addressed non-malignant outcomes. Meanwhile, the specialized Cluster #13 (tracheal bronchus) and Cluster #14 (airway microbiome) represented emerging frontiers; the latter demonstrated particularly strong integration with mechanistic and clinical research. Collectively, these clusters depicted an interconnected research continuum spanning environmental exposures, biological mechanisms, clinical manifestations, and public health responses.

## 4 Discussion

Our analysis outlines a dynamic and rapidly evolving research landscape at the intersection of lung cancer and air pollution, which has grown increasingly interdisciplinary. The field has progressed from an initial focus on establishing straightforward epidemiological associations to a more complex paradigm integrating exposure science, molecular biology, computational analytics, and global public health. This evolution, reflected in bibliometric trends across publications, citations, collaborations, and keyword dynamics, is characterized by three interconnected shifts: the diversification of studied pollutants and exposure scenarios, a deepening of mechanistic inquiry from organ to molecular levels, and the integration of novel methodologies to address emerging global health challenges. The following discussion interprets these bibliometric trends within the broader context of environmental health science.

### 4.1 Etiological spectrum: expanding from indoor and occupational hazards to ambient air pollution

Analysis of keyword co-occurrence and evolution reveals a clear temporal shift in research priorities, tracing a distinct trajectory in the understanding of lung cancer etiology. Publication and citation trends, together with the progression of keyword clusters from foundational to contemporary themes, illustrate this transition. Initially, the field concentrated on establishing definitive links between specific, high-intensity exposures and lung carcinogenesis. Seminal studies, frequently cited and forming early citation bursts, firmly established active and passive tobacco smoke as leading causes of lung cancer mortality (37). Substantial evidence, reflected in early keyword clusters including “Radon” and “Asbestos,” confirmed that indoor air pollutants, especially residential exposure to radon, thoron, and their decay products, constituted a major risk factor for lung cancer (38, 39). Concurrently, a robust body of evidence from occupational settings identified another pillar of traditional risk. Landmark case-control studies demonstrated that inhalation of specific occupational hazards, including asbestos, chrysotile, and silica, significantly increases the risk of lung cancer and other respiratory diseases (40). Research during this period, as indicated by the historical distribution of publications and cited references, primarily addressed discrete, often point-source exposures.

A significant paradigm shift, evident in the surge of publications after 2015 and the growing prominence of keywords such as “PM2.5” and “Air Pollutants,” occurred as research broadened to address complex mixtures of ambient air pollution, with a predominant focus on particulate matter. The pervasive threat of PM_2.5_ and its constituents, notably black carbon and ultrafine particles, has been conclusively linked to elevated lung cancer risk through mechanisms involving oxidative stress and DNA damage (30). This expansion of research, mirrored in the increasing volume of multi-country publications and international collaborations, has fundamentally redefined the scope of environmental lung carcinogens. It marks a transition from investigating individual-level risks from specific hazards to assessing population-level health impacts from ubiquitous environmental exposures. Furthermore, the recent convergence of this field with global health challenges, highlighted by the emergence of keywords such as “Covid-19,” is exemplified by studies examining interactions between chronic PM_2.5_ exposure and COVID-19 outcomes (41). This trend underscores a growing imperative, reflected in recent citation bursts and research fronts, to understand the syndemic effects of air pollution in the context of emerging respiratory pandemics.

### 4.2 Molecular pathogenesis: from epidemiological associations to mechanistic insights and precision prevention

A second major trend, discernible from the keyword co-occurrence network showing dense clusters around “Oxidative Stress,” “Gene Expression,” and “DNA damage,” as well as from thematic clusters identified by CiteSpace, represents a paradigm shift from observational associations toward a multilevel mechanistic understanding of lung carcinogenesis. Although traditional epidemiological studies linking environmental and occupational exposures to lung cancer incidence remain fundamental, the research frontier has expanded considerably to decipher the biological pathways underlying these associations. This shift is bibliometrically evidenced by the growing frequency and centrality of keywords related to DNA damage, oxidative stress, inflammation, and genotoxicity within the analytical network, reflecting a concerted effort to elucidate the molecular and cellular events driving cancer initiation and promotion.

Recent mechanistic research, often published in high-impact journals identified in the co-citation network, has provided profound insights into how specific components of air pollution, particularly PM_2.5_ and PAHs, drive lung carcinogenesis at the molecular level. The mechanisms of PM_2.5_ extend beyond generalized oxidative stress. Emerging evidence from experimental models indicates that PM_2.5_ exposure induces epigenetic reprogramming, notably through the alteration of DNA methylation patterns in genes critical for cell cycle control and DNA repair (42). Furthermore, studies demonstrate that chronic exposure to PM_2.5_ can inhibit the activity of key DNA repair enzymes, creating a permissive environment for the accumulation of persistent DNA damage and genomic instability (43, 44). Concurrently, research on PAHs, key constituents of PM_2.5_, reveals their multifaceted attack on cellular integrity. The classical pathway involves metabolic activation of prototypical PAHs including benzo[a]pyrene into highly reactive diol epoxides, which form bulky DNA adducts that distort the DNA helix and provoke mutation-prone replication (45). Simultaneously, specific PAHs can act as ligands for the aryl hydrocarbon receptor (AhR), triggering its translocation to the nucleus and initiating the transcription of numerous genes involved in xenobiotic metabolism. This process amplifies their metabolic activation and sustains a cycle of oxidative stress and pro-inflammatory cytokine production. This chronic inflammation, characterized by persistent activation of NF-κB and NLRP3 inflammasome pathways, fosters a tumor-promoting microenvironment (46). Collectively, these findings delineate a convergent pathogenic model in which PM_2.5_ and its constituent PAHs cooperatively induce genetic mutations through DNA adduct formation, disrupt epigenetic regulation, and foster a pro-tumorigenic inflammatory milieu, thereby providing a comprehensive molecular basis for the established epidemiological link between air pollution and lung cancer.

Building on these mechanistic discoveries, biomarkers of exposure, effect, and susceptibility now form a critical bridge connecting external pollution to internal biological responses. Research now systematically characterizes specific biomarkers, ranging from PAH-DNA adducts and 8-hydroxy-2’-deoxyguanosine (8-OHdG) for exposure and oxidative damage, to emerging epigenetic signatures illustrated by DNA methylation patterns in repetitive elements or promoter regions of tumor suppressor genes (47–49). This approach not only validates exposure at the individual level but also captures early preclinical biological effects, enabling the identification of susceptible populations based on genetic predispositions and epigenetic profiles.

### 4.3 Methodological evolution: advancing from analytical techniques to global burden assessment

A third major shift, reflected in the bibliometric data through the emergence of keywords such as “Machine Learning” and “Global Burden,” along with citation bursts of large-scale burden studies, is both methodological and perspectival, fundamentally transforming research methodologies and interpretive frameworks. The rigorous synthesis of evidence through systematic reviews and meta-analyses has become standard practice for establishing causal inference and quantifying pooled effect estimates, a trend supported by the high citation counts of such seminal reviews. A 2014 systematic review and meta-analysis in Environmental Health Perspectives quantitatively synthesized epidemiological evidence, revealing that each 10-μg/m³ increase in PM_2.5_ exposure was associated with a 9% elevated risk of lung cancer, thereby providing the crucial quantitative foundation for the International Agency for Research on Cancer’s classification of particulate matter as a Group I carcinogen (50). Simultaneously, machine learning integration, a recent hotspot in keyword analysis, has introduced a paradigm shift in exposure assessment and risk prediction. Utilizing data from 203 countries between 1990 and 2019, a 2023 study employed advanced ensemble classifiers to achieve high-precision predictions of lung cancer mortality, pinpointing CO as a critical feature and validating machine learning’s role in assessing global air pollution health risks (51).

This methodological advancement enables more precise quantification of public health impacts across multiple scales. The bibliometric focus on terms including burden of disease, global burden of disease, and disease burden analyses, particularly in highly cited recent papers, highlights a commitment to translating research findings into actionable metrics for policy intervention. For example, a 2023 global burden of disease study published in The Lancet Planetary Health specifically quantified the global burden of lung cancer attributable to ambient PM_2.5_ pollution, revealing substantial regional variations and identifying key demographic groups bearing the highest disease burden. These analyses extend beyond mortality statistics by incorporating metrics such as disability-adjusted life years, providing a more comprehensive assessment of the health loss attributable to specific air pollution sources, from household coal combustion to traffic-related emissions (52). These findings validate global burden estimates while providing crucial insights for developing targeted interventions, demonstrating how localized mechanistic studies can inform global public health actions.

## 5 Limitations

As the first bibliometric analysis to specifically examine the nexus between air pollution and lung cancer, this study maps the research landscape comprehensively; however, it is not without limitations. First, although relying solely on WoSCC and Scopus is standard practice in bibliometric research, this approach may have omitted relevant studies indexed in other databases (e.g., PubMed, CNKI). Second, the restriction to English-language articles and the selected start date of 1990 could introduce language bias and exclude seminal non-English or earlier works, thereby potentially compromising the historical comprehensiveness of the research mapping. And our analysis is based on a snapshot of the literature up to 2025. The continuing accrual of citations and future database updates will inevitably refine specific rankings, particularly for recent publications. However, the major historical trends and structural patterns identified in this study are expected to remain valid. Third, bibliometric methods are inherently quantitative and do not incorporate qualitative assessments of publication quality; therefore, identifying the most scientifically rigorous works falls beyond the scope of this approach. Despite these limitations, this analysis successfully delineates the developmental trajectory and emerging themes in air pollution-lung cancer research, thereby establishing a foundational reference for understanding the field’s current state and future directions. It should be noted that bibliometric indicators such as citation counts are influenced by multiple factors, including journal visibility and publication language. Here, we interpret these indicators as reflections of scholarly attention and influence within the global research community, not as direct measures of scientific quality or societal impact.

## 6 Conclusion

Our analysis delineates the dynamic evolution and intellectual structure of research on the association between air pollution and lung cancer. The study makes several key contributions: it maps the field’s rapid growth and shifting publication trends; identifies leading contributors, naming China and the United States as the most productive nations and Harvard University and the Chinese Academy of Sciences as the most active institutions; reveals core research themes through keyword co-occurrence, centering on “Particulate Matter” and “Lung Cancer”; and uncovers a paradigm shift from classical exposure assessment toward mechanistic investigations and machine learning applications. Future research directions will likely prioritize three key areas: advancing mechanistic understanding of particulate matter-induced carcinogenesis, integrating comprehensive exposome-wide assessments with genomic susceptibility analyses, and developing innovative real-time exposure monitoring technologies. Furthermore, fostering interdisciplinary collaborations among epidemiology, molecular toxicology, and data science will be essential to advancing precision prevention strategies and evidence-based policy interventions. These efforts will ultimately aim to mitigate the global burden of air pollution-related lung cancer and improve public health outcomes.

## Data Availability

The raw data supporting the conclusions of this article will be made available by the authors, without undue reservation.

## Supplementary material

Supplementary Table 1 Most Relevant Countries by Corresponding Author Contributions (1990-2025).

Supplementary Table 2 TOP 10 Affiliations and Article Counts on Air Pollution and Lung Cancer (1990-2025).

Supplementary Table 3 Bibliometric Overview of Air Pollution and Lung Cancer (1990-2025).
