# Supplementary material for: Mapping three decades of air pollution–lung cancer research: trends, hotspots, and networks (1990-2025)
Source: Front Oncol. 2025 Dec 18;15:1698246. doi: 10.3389/fonc.2025.1698246 (PMC12756124; doi:10.3389/fonc.2025.1698246)
Supplement: Supplementary Table 1 — Most Relevant Countries by Corresponding Author Contributions (1990-2025). [file Table1.docx]

**Supplemental Tables**

**Supplementary Table 1.** Most Relevant Countries by Corresponding Author Contributions (1990-2025)

**Supplementary Table 2.** TOP 10 Affiliations and Article Counts on Air Pollution and Lung Cancer (1990-2025)

**Supplementary Table 3.** Bibliometric Overview of Air Pollution and Lung Cancer (1990-2025)

**Supplementary Table 1. Most Relevant Countries by Corresponding Author Contributions**

| Rank | **Country** | **Articles** | **Articles %** | **SCP** | **MCP** | **MCP %** |
| --- | --- | --- | --- | --- | --- | --- |
| 1 | CHINA | 412 | 9.7 | 304 | 108 | 26.2 |
| 2 | USA | 291 | 6.9 | 176 | 115 | 39.5 |
| 3 | KOREA | 108 | 2.5 | 104 | 4 | 3.7 |
| 4 | ITALY | 84 | 2 | 55 | 29 | 34.5 |
| 5 | INDIA | 71 | 1.7 | 55 | 16 | 22.5 |
| 6 | CANADA | 60 | 1.4 | 32 | 28 | 46.7 |
| 7 | UNITED KINGDOM | 57 | 1.3 | 33 | 24 | 42.1 |
| 8 | IRAN | 45 | 1.1 | 32 | 13 | 28.9 |
| 9 | FRANCE | 38 | 0.9 | 20 | 18 | 47.4 |
| 10 | SPAIN | 32 | 0.8 | 17 | 15 | 46.9 |
| 11 | NETHERLANDS | 31 | 0.7 | 9 | 22 | 71 |
| 12 | AUSTRALIA | 27 | 0.6 | 12 | 15 | 55.6 |
| 13 | DENMARK | 25 | 0.6 | 16 | 9 | 36 |
| 14 | BRAZIL | 23 | 0.5 | 17 | 6 | 26.1 |
| 15 | JAPAN | 23 | 0.5 | 15 | 8 | 34.8 |
| 16 | POLAND | 23 | 0.5 | 18 | 5 | 21.7 |
| 17 | GERMANY | 21 | 0.5 | 14 | 7 | 33.3 |
| 18 | GREECE | 18 | 0.4 | 9 | 9 | 50 |
| 19 | MEXICO | 18 | 0.4 | 14 | 4 | 22.2 |
| 20 | SWEDEN | 18 | 0.4 | 11 | 7 | 38.9 |
| 21 | THAILAND | 17 | 0.4 | 12 | 5 | 29.4 |
| 22 | PORTUGAL | 13 | 0.3 | 10 | 3 | 23.1 |
| 23 | TURKEY | 11 | 0.3 | 8 | 3 | 27.3 |
| 24 | NORWAY | 10 | 0.2 | 8 | 2 | 20 |
| 25 | SAUDI ARABIA | 10 | 0.2 | 5 | 5 | 50 |

**Supplementary Table 2. Most Relevant Affiliations by Corresponding Author Contributions**

| Affiliation | Articles |
| --- | --- |
| HARVARD UNIVERSITY | 146 |
| UTRECHT UNIVERSITY | 115 |
| CHINESE ACADEMY OF SCIENCES | 106 |
| UNIVERSITY OF CALIFORNIA SYSTEM | 105 |
| IMPERIAL COLLEGE LONDON | 86 |
| NATIONAL INSTITUTES OF HEALTH (NIH) - USA | 79 |
| NIH NATIONAL CANCER INSTITUTE (NCI) | 74 |
| HARVARD T.H. CHAN SCHOOL OF PUBLIC HEALTH | 73 |
| UNIVERSITY OF LONDON | 71 |
| HEALTH CANADA | 68 |

**Supplementary Table 3. Bibliometric Overview of Air Pollution and Lung Cancer (1990-2025)**

| **Description** | **Results** |
| --- | --- |
| MAIN INFORMATION ABOUT DATA |  |
| Timespan | 1990:2025 |
| Sources (Journals, Books, etc) | 1063 |
| Documents | 4238 |
| Annual Growth Rate % | 6.35 |
| Document Average Age | 9.91 |
| Average citations per doc | 58.42 |
| References | 180809 |
| DOCUMENT CONTENTS |  |
| Keywords Plus (ID) | 18174 |
| Author's Keywords (DE) | 7125 |
| AUTHORS |  |
| Authors | 16823 |
| Authors of single-authored docs | 272 |
| AUTHORS COLLABORATION |  |
| Single-authored docs | 320 |
| Co-Authors per Doc | 6.53 |
| International co-authorships % | 13.1 |
| DOCUMENT TYPES |  |
| article | 3960 |
| review | 278 |
