# Supplementary material for: Mapping three decades of air pollution–lung cancer research: trends, hotspots, and networks (1990-2025)
Source: Front Oncol. 2025 Dec 18;15:1698246. doi: 10.3389/fonc.2025.1698246 (PMC12756124; doi:10.3389/fonc.2025.1698246)
Supplement: Supplementary Table 2 — TOP 10 Affiliations and Article Counts on Air Pollution and Lung Cancer (1990-2025). [file Table2.docx]

**Search Strategy for Bibliometric Analysis of Air Pollution**

**and Lung Cancer Literature**

| Wed of Science | | |
| --- | --- | --- |
| #1 | TI=("Air Pollution*" OR "Pollution, Air" OR "Air Quality" OR "Air Pollutant*" OR "Pollutants, Air" OR “Pollutant, Air" OR "Environmental Pollution" OR "Pollution, Environmental" OR "Air Pollutants, Environmental" OR "Environmental Pollutants, Air" OR "Air Environmental Pollutants" OR "Pollutants, Air Environmental" OR "Environmental Air Pollutants")) OR AB=("Air Pollution*" OR "Pollution, Air" OR "Air Quality" OR "Air Pollutant*" OR "Pollutants, Air" OR “Pollutant, Air" OR "Environmental Pollution" OR "Pollution, Environmental" OR "Air Pollutants, Environmental" OR "Environmental Pollutants, Air" OR "Air Environmental Pollutants" OR "Pollutants, Air Environmental" OR "Environmental Air Pollutants")) OR AK=("Air Pollution*" OR "Pollution, Air" OR "Air Quality" OR "Air Pollutant*" OR "Pollutants, Air" OR “Pollutant, Air" OR "Environmental Pollution" OR "Pollution, Environmental" OR "Air Pollutants, Environmental" OR "Environmental Pollutants, Air" OR "Air Environmental Pollutants" OR "Pollutants, Air Environmental" OR "Environmental Air Pollutants") |  |
| #2 | TI=("Neoplasm, Lung" OR "Neoplasms, Pulmonary" OR "Neoplasm, Pulmonary" OR "Lung Neoplasm" OR "Lung Neoplasms" OR "Neoplasm, Lung" OR "Lung Cancer" OR "Cancer, Lung" OR "Cancers, Lung" OR "Lung Cancers" OR "Cancer of Lung" OR "Pulmonary Cancer" OR "Cancer, Pulmonary" OR "Cancers, Pulmonary" OR "Pulmonary Cancers" OR "Cancer of the Lung") OR AK=("Neoplasm, Lung" OR "Neoplasms, Pulmonary" OR "Neoplasm, Pulmonary" OR "Lung Neoplasm" OR "Lung Neoplasms" OR "Neoplasm, Lung" OR "Lung Cancer" OR "Cancer, Lung" OR "Cancers, Lung" OR "Lung Cancers" OR "Cancer of Lung" OR "Pulmonary Cancer" OR "Cancer, Pulmonary" OR "Cancers, Pulmonary" OR "Pulmonary Cancers" OR "Cancer of the Lung") OR AB=("Neoplasm, Lung" OR "Neoplasms, Pulmonary" OR "Neoplasm, Pulmonary" OR "Lung Neoplasm" OR "Lung Neoplasms" OR "Neoplasm, Lung" OR "Lung Cancer" OR "Cancer, Lung" OR "Cancers, Lung" OR "Lung Cancers" OR "Cancer of Lung" OR "Pulmonary Cancer" OR "Cancer, Pulmonary" OR "Cancers, Pulmonary" OR "Pulmonary Cancers" OR "Cancer of the Lung") |  |
| #3 | #1 AND #2 | 1910 |

| Scopus | | |
| --- | --- | --- |
| #1 | TITLE-ABS-KEY (“Lung Neoplasms” OR “Neoplasm, Lung" OR "Neoplasms, Pulmonary" OR "Neoplasm, Pulmonary" OR "Lung Neoplasm" OR "Lung Neoplasms" OR "Neoplasm, Lung" OR "Lung Cancer" OR "Cancer, Lung" OR "Cancers, Lung" OR "Lung Cancers" OR "Cancer of Lung" OR "Pulmonary Cancer" OR "Cancer, Pulmonary" OR "Cancers, Pulmonary" OR "Pulmonary Cancers" OR "Cancer of the Lung") |  |
| #2 | TITLE-ABS-KEY (“Air Pollutants” OR "Air Pollution*" OR "Pollution, Air" OR "Air Quality" OR "Air Pollutant*" OR "Pollutants, Air" OR “Pollutant, Air" OR "Environmental Pollution" OR "Pollution, Environmental" OR "Air Pollutants, Environmental" OR "Environmental Pollutants, Air" OR "Air Environmental Pollutants" OR "Pollutants, Air Environmental" OR "Environmental Air Pollutants") |  |
| #3 | #1 AND #2 | 5816 |
